# Medical Obituary

**Published:** 1833

**Authors:** 


### Art. XX. Medical Obituary.

*John Woolley, M. D.* We neglected to mention in our last number, the death of this gentleman, for many years a respectable physician and citizen of this place. He was a graduate of the first class of the Medical College of Ohio, in the spring of 1821, For many years he was one of the Censors of the First District Society; and in the winter of 1827–8, acted as president of the Medical Convention of the State.

*Thomas Sargent, M. D.* of Apoplexy, on the 29th December. Dr. S. was an emigrant from Philadelphia. He united in himself the clerical and medical professions. The fatal attack seized him in the pulpit of one of the Methodist Episcopal churches of this city. He was a good physician, an amiable man, and a zealous and exemplary Christian.

*William Gardner, M. D.*, of the Western Reserve, in this State. We have had some correspondence with this gentleman, and were led to regard him as a highly-respectable member of the profession. We hope some of his friends will furnish us with a condensed obituary.

